# Multi-stage tomato fruit recognition method based on improved YOLOv8

**DOI:** 10.3389/fpls.2024.1447263

**Published:** 2024-09-05

**Authors:** Yuliang Fu, Weiheng Li, Gang Li, Yuanzhi Dong, Songlin Wang, Qingyang Zhang, Yanbin Li, Zhiguang Dai

**Affiliations:** ^1^ North China University of Water Resources and Electric PowerSchool of Water Conservancy, Zhengzhou, China; ^2^ State Key Laboratory of Eco-Hydraulics in the Northwest Arid Region of China, Xi’an University of Technology, Xi’an, Shanxi, China; ^3^ School of Water Conservancy, North China University of Water Resources and Electric Power, Zhengzhou, Henan, China; ^4^ Henan University of Science and Technology College of Agricultural Engineering, Luoyang, China

**Keywords:** image recognition, object detection, YOLOv8, EfficientViT, auxiliary detection head, tomato

## Abstract

**Introduction:**

In the field of facility agriculture, the accurate identification of tomatoes at multiple stages has become a significant area of research. However, accurately identifying and localizing tomatoes in complex environments is a formidable challenge. Complex working conditions can impair the performance of conventional detection techniques, underscoring the necessity for more robust methods.

**Methods:**

To address this issue, we propose a novel model of YOLOv8-EA for the localization and identification of tomato fruit. The model incorporates a number of significant enhancements. Firstly, the EfficientViT network replaces the original YOLOv8 backbone network, which has the effect of reducing the number of model parameters and improving the capability of the network to extract features. Secondly, some of the convolutions were integrated into the C2f module to create the C2f-Faster module, which facilitates the inference process of the model. Third, the bounding box loss function was modified to SIoU, thereby accelerating model convergence and enhancing detection accuracy. Lastly, the Auxiliary Detection Head (Aux-Head) module was incorporated to augment the network's learning capacity.

**Result:**

The accuracy, recall, and average precision of the YOLOv8-EA model on the self-constructed dataset were 91.4%, 88.7%, and 93.9%, respectively, with a detection speed of 163.33 frames/s. In comparison to the baseline YOLOv8n network, the model weight was increased by 2.07 MB, and the accuracy, recall, and average precision were enhanced by 10.9, 11.7, and 7.2 percentage points, respectively. The accuracy, recall, and average precision increased by 10.9, 11.7, and 7.2 percentage points, respectively, while the detection speed increased by 42.1%. The detection precision for unripe, semi-ripe, and ripe tomatoes was 97.1%, 91%, and 93.7%, respectively. On the public dataset, the accuracy, recall, and average precision of YOLOv8-EA are 91%, 89.2%, and 95.1%, respectively, and the detection speed is 1.8 ms, which is 4, 4.21, and 3.9 percentage points higher than the baseline YOLOv8n network. This represents an 18.2% improvement in detection speed, which demonstrates good generalization ability.

**Discussion:**

The reliability of YOLOv8-EA in identifying and locating multi-stage tomato fruits in complex environments demonstrates its efficacy in this regard and provides a technical foundation for the development of intelligent tomato picking devices.

## 1 Introduction

Tomatoes, with their rich nutrients and unique flavor, are highly favored by consumers. As market demand continues to grow, so too does the production and cultivation scale of tomatoes (Su et al., 2022). Currently, the harvesting process still relies on manual labor which is subject to personal judgment and past experience, leading to issues such as low efficiency, high costs, and untimely harvesting (Han et al., 2022; Yang et al., 2024). The use of intelligent robotic harvesters to replace human labor in tomato picking holds significant importance and prospects for the modernization of the tomato industry. Given that tomatoes have a short ripening period and are not easy to store, it is necessary to screen tomatoes at different maturity stages according to actual needs; this plays a positive role in increasing farmers’ income (Nassiri et al., 2022). The basic requirement for achieving intelligent harvesting lies in accurately identifying and locating multi-stage tomato fruits – a key step towards implementing precision agriculture (Bai et al., 2023; Lin et al., 2024). Therefore, enhancing model detection performance is crucial for realizing the automation of tomato harvesting.

Traditional image processing methods extract features such as color, shape, and texture from images by analyzing high-resolution pictures and designing artificial features to match and recognize target fruits. However, these methods have limitations in automatic feature extraction, detection speed, and accuracy (Wang et al., 2022). They are susceptible to environmental influences and the number of fruit colors, lacking reliability and robustness in complex scenarios, which makes it difficult to meet practical demands (Zhang et al., 2023a). With the continuous development of machine vision technology, Convolutional Neural Networks (CNN) show enormous potential in agriculture due to their rapid processing capabilities and adaptability to complex scenes. The current mainstream algorithms are divided into two categories: a second-order detection algorithm based on candidate regions represented by the R-CNN series; and a first-order monitoring algorithm based on network regression represented by the YOLO series. Long Jiehua et al. (Long et al., 2021) proposed an improved Mask R-CNN model that provides a basis for detecting maturity levels of tomatoes and intelligent picking operations. Mu et al. (Mu et al., 2020) integrated Faster R-CNN with transfer learning for detecting unripe tomato fruits. Li Tianhua (Li et al., 2021) et al. proposed a recognition method that fuses YOLOv4 with HSV to segment red areas on tomatoes; however, this approach does not perform well when multiple fruits overlap one another. Zeng et al. (Zeng et al., 2023) reconstructed the backbone network of YOLOv5 using lightweight Bneck modules they also pruned it which resulted in a 78% reduction in model parameters and an 84.15% decrease floating-point operations per second leading greatly increased detection efficiency though its efficiency at spotting ripe tomatoes was lower. Liu Fang (Liu et al., 2020) and others proposed the multi-scale IMS-YOLO, which achieves a tomato detection accuracy of 97.13% in complex greenhouse environments, but performs poorly in detecting small objects. Zhang Junning (Zhang et al., 2023b) integrated the CBAM attention mechanism into the YOLOv5s network to give more focus on green tomatoes, enhancing the recognition accuracy of two types of tomatoes. Similarly, (Appe et al., 2023) replaces the DIoU loss function on this basis and achieves an average detection accuracy of up to 88.1% for overlapping targets and small target tomatoes. Gao (Gao et al., 2024) proposed an improved Soft-NMS algorithm for improving YOLOv5s by taking into account the real-time condition of the picking robot in continuous working condition, which significantly improves the recognition of tomato in continuous working. Miao Ronghui (Miao et al., 2023) and others adopted an improved YOLOv7 model to detect multistage cherry tomatoes, effectively reducing the amount of model parameters and memory usage while speeding up inference. Chen et al. (Chen et al., 2024a) proposed the MTD-YOLOv7 model, used for multitask maturity detection of cherry tomato bunches and fruits, achieving a detection accuracy of 86.6% and an inference speed of 4.9ms, demonstrating outstanding performance. Based on information mapping and morphological operations, the SimAM attention module and MobileNeXt are integrated into YOLOv7-tiny, while the improved DeepSORT algorithm is integrated to propose a real-time detection algorithm for multiple maturity tomatoes with good results (Meng et al., 2024).

Recently, many scholars have also considered deploying the improved YOLO algorithm on edge devices for tasks such as tomato fruit morphology recognition (Du et al., 2023; Fu et al., 2024), pest and disease dynamics detection (Jin et al., 2024; Wang and Liu, 2024), and growth monitoring (Chen et al., 2024b; Tian et al., 2024), and its excellent task completion performance demonstrates notable competitiveness.

The above research demonstrates the feasibility and potential application of deep learning-based multi-stage target detection for tomatoes, but the following issues still exist: Fruits and small targets that are obscured may be missed or incorrectly identified; the model structure is complex and has a large number of parameters, leading to redundant feature extraction; under complex environments, detection efficiency and accuracy are relatively low. Based on this, the paper proposes an improved YOLOv8 model aimed at efficiently recognizing tomatoes at different growth stages in complex greenhouse environments. By reducing the number of parameters and optimizing network structure, a balance between model accuracy and efficiency is achieved.

## 2 Materials and methods

### 2.1 Data collection and preprocessing

#### 2.1.1 Data collection

**Figure 1:**
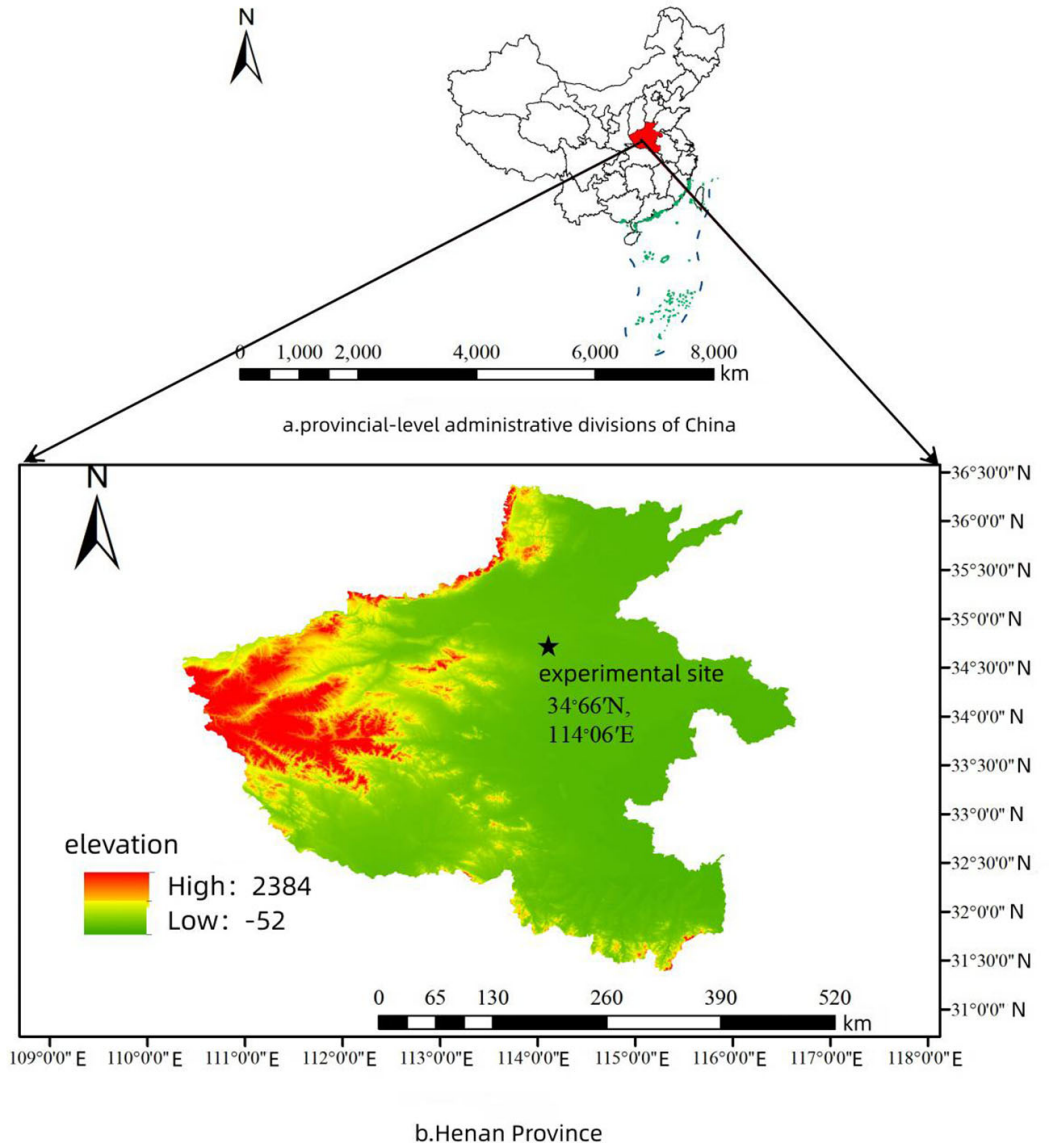
Data collection point location (34.66°N, 114.06°E).

The data collection site is located at the Yuzhong Greenhouse Complex in Zhongmu County, Zhengzhou City, Henan Province, China (34.66°N, 114.06°E), As shown in **Figure 1**. focusing on tomatoes cultivated on greenhouse ridges. This study selected the locally representative “YingFen-No.58” variety of tomatoes as the research subject and used an EOS M50 Mark II camera to take photographs from December 14 to 27, 2023, between 9:00 AM and 5:00 PM.

**Figure 2:**
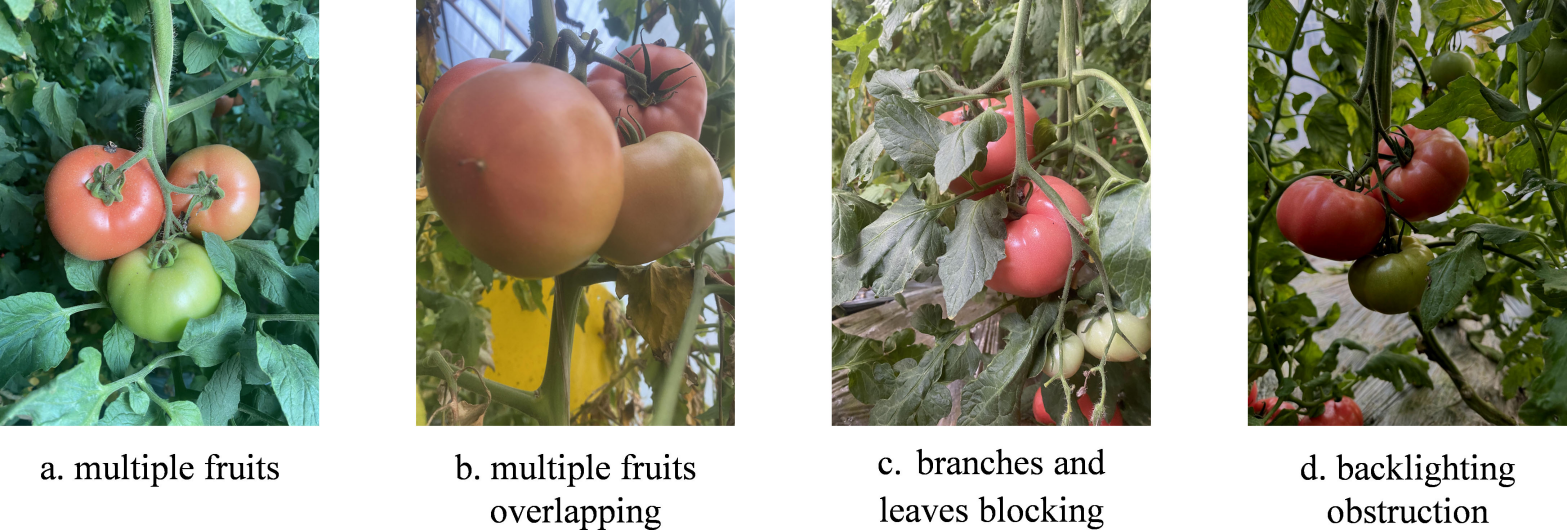
Sample image collection. **(A)** multiple fruits. **(B)** multiple fruits overlapping. **(C)** branches and leaves blocking. **(D)** backlighting obstruction.

To enhance the model’s generalization ability and diversify the dataset, we seek to downplay structured features of greenhouses. Batches of tomato plants were photographed in their natural environment in the greenhouse, taking into account different time periods, densities, shading conditions, light conditions, and other actual picking conditions in the sampling process. After screening, 716 high-resolution images (3024 pixels x 4032 pixels) were obtained. **Figure 2** Sample image collection displays some images from the dataset.

#### 2.1.2 Data preprocessing

This study utilizes Roboflow to annotate the collected raw images, accurately delineating the contours of the fruit using minimal bounding rectangles to ensure each box contains only one piece of fruit and minimizes background noise. Do not label fruit that is severely obscured or relatively small. According to the experience of local farmers “Ripe-Tomato” (Ripe tomatoes in bright red)、”Semi-ripe Tomato” (light orange-yellow semi-ripe tomato)、”Unripe-Tomato” (Green unripe tomatoes) Three categories. After the annotation is complete, use the built-in scaling feature to process the image, uniformly adjusting the resolution of the image to 640 pixels × 640 pixels. Save this as a.txt file. The stored information includes: target category, coordinates of the bounding box center point, and dimensions such as width and height.

**Figure 3:**
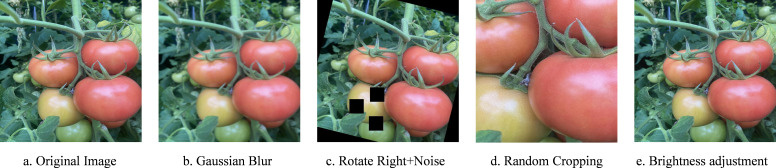
Effects of data augmentation. **(A)** Original Image. **(B)** Gaussian Blur. **(C)** Rotate Right+Noise. **(D)** Random Cropping. **(E)** Brightness adjustment.

Divide the dataset randomly into training, validation, and test sets in a 7:2:1 ratio. To enhance the model’s robustness and its ability to resist interference, as well as to avoid overfitting, the training set was augmented using Roboflow tools through methods such as Gaussian blur and random cropping. As shown in **Figure 3**, each original image generated four new images, resulting in a total of 2720 images.

### 2.2 Construction of experimental platform and parameter settings

The operating system used for the experiment is Linux, with an Intel(R) Core(TM) i7–10750H CPU @ 2.60GHz, NVIDIA GeForce RTX3080Ti GPU, 32GB of RAM, and a 500GB HDD. The programming language is Python 3.9, utilizing the Pytorch 1.9 deep learning framework with CUDA 11.8 GPU acceleration. The initial learning rate is set to 0.01, momentum parameter to 0.937, iteration rounds to 300, target class number to 3, and Batch_size to 32.

### 2.3 YOLOv8 network model

YOLOv8 is the latest SOTA (State-of-the-Art) model released by the Ultralytics team in 2023. Building on the success of YOLOv5, it incorporates new improvements and features to further enhance flexibility and performance. The main changes include: replacing the original C3 module with the C2f module; removing the convolution operation in the upsampling process; introducing a new anchor-free decoupled head structure. The network structure of YOLOv8 includes the backbone network, neck network, and head network. The backbone network adopts the Darknet53 structure, obtaining features of different sizes through five down-samplings. The C3 module has been replaced with a more abundant C2f module to increase branches for enriched gradient backpropagation. The neck network utilizes a PANet, enhancing the receptive field and improving feature fusion capabilities by bidirectional integration of dual-layer features. The head network adopts an Anchor Free strategy and a decoupled head structure, using a parallel branch architecture to separate positioning from classification tasks while discarding confidence prediction to accelerate model convergence.

Although the YOLOv8 model belongs to the latest iteration of the YOLO series, it still has some limitations. For example, the low resolution of the feature map due to the restricted working conditions of the actual scene makes it perform poorly during small target detection; furthermore, despite its highly efficient structural design, real-time processing on resource-constrained devices is still challenging; and lastly, its sensitivity to occlusion and lighting variations also affects its robustness and reliability in practical applications.

#### 2.3.1 Improvement of the network model

**Figure 4:**
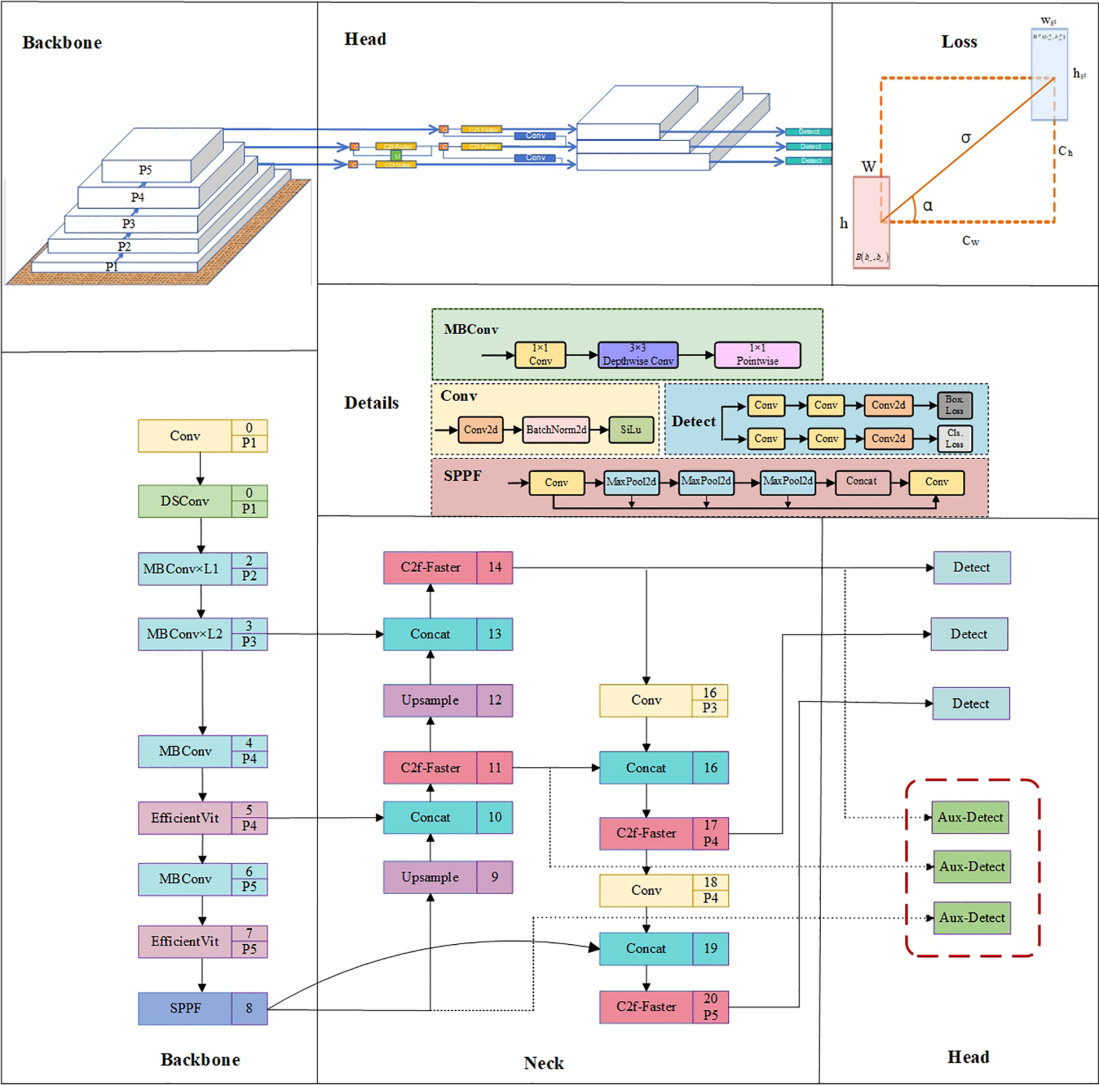
YOLOv8-EA network architecture diagram. Conv represents ordinary convolution operation; MBConv represents convolution with inverted residual structure; C2f-Faster introduces a C2f module with partial convolution; Upsample refers to upsampling; Contact denotes concatenation operation; SPPF stands for fast pooling pyramid module; Aux-Detect is an auxiliary detection head, called only during training; Bbox.Loss and Cls.Loss stand for bounding box loss and classification loss, respectively.

The improved network structure of YOLOv8-EA, as shown in **Figure 4**, utilizes EfficientViT as the backbone network. This version incorporates variable convolutions into the original C2f module, switches to SIoU loss function, and adds Aux-Detect. These enhancements aim to further strengthen the model’s ability to capture key features in complex environments, reduce false negatives and false positives, and enhance the robustness of the algorithm in detecting tomato fruits under challenging conditions.

a) EfficientViT Network

**Figure 5:**
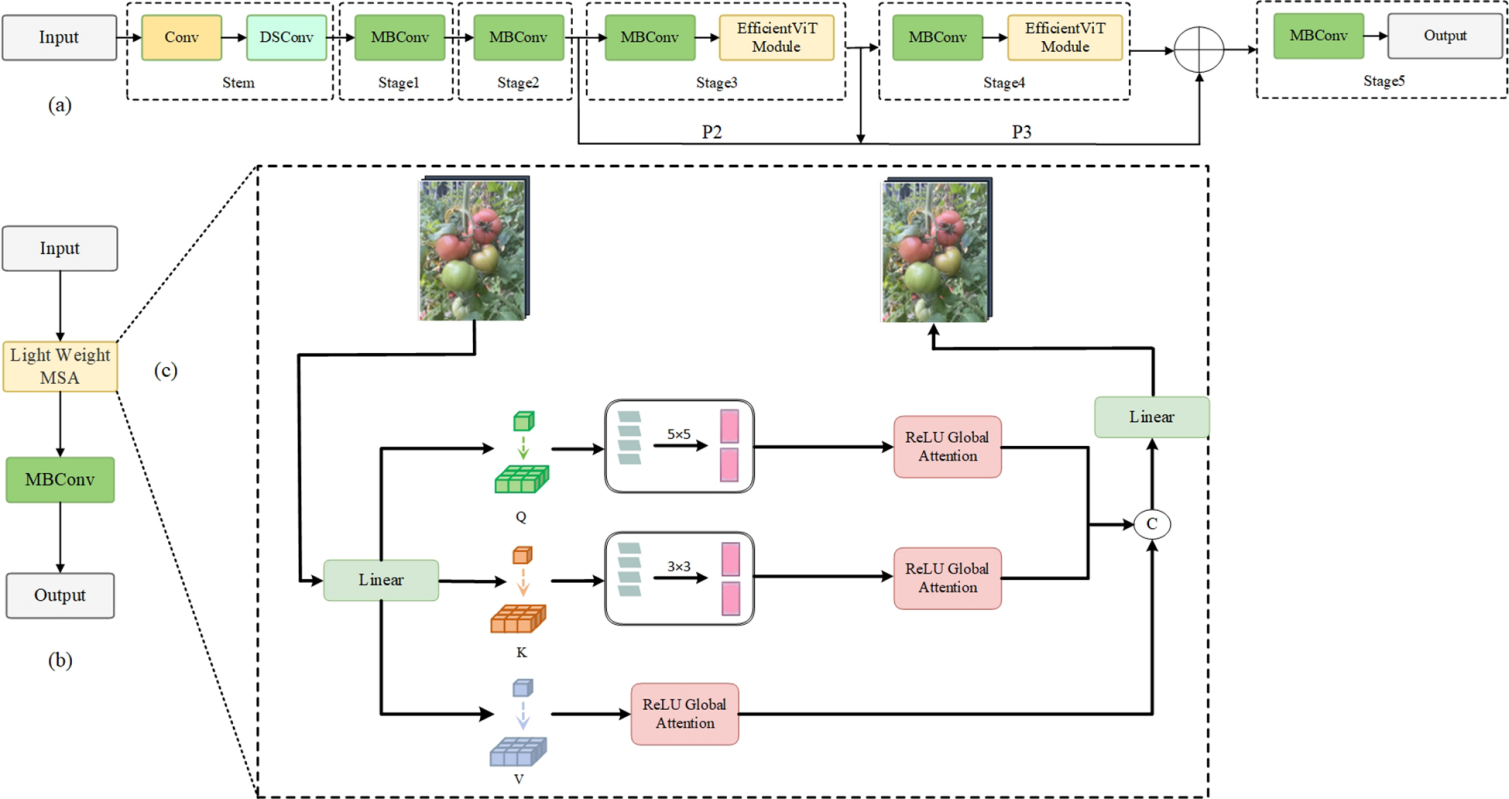
EfficientViT network architecture diagram.

EfficientViT (Cai et al., 2023) (Efficient Vision Transformer) is a variant network model based on the Transformer architecture, facilitating efficient deployment and real-time inference computing of ViT (Vision Transformer) on edge devices. As shown in **Figure 5**, EfficientViT employs linear attention in place of softmax attention, enhancing the ability to extract local features via deep convolution; it uses ReLU linear attention to achieve a global view while reducing complexity and maintaining the capability to extract both local and global features.

The EfficientViT structure is shown in **Figure 5A**, and the core building block “EfficientViT Module” is shown in **Figure 5B**. This module consists of a Lightweight MSA (Geser et al., 2006) module (as shown in **Figure 5C**) and an MBConv module. The lightweight MSA module uses linear projection layers to extract Q, K, V tokens, and uses small convolution kernels for information aggregation to form multi-scale tokens. By employing a global self-attention mechanism based on ReLU, each scale feature is weighted to capture information at various scales. Subsequently, the outputs are concatenated and sent to the final linear projection layer for feature fusion, producing more expressive and diverse global features. This model introduces a method that enhances the ability to learn globally across multiple scales by aggregating nearby Q, K, and V values in order to reduce computational and storage costs while using small convolutional kernels to achieve a balance between accuracy and efficiency. Meanwhile, the MBConv module enhances gradient propagation characteristics to better capture local information (Nascimento et al., 2019).

Assuming the input is, the self-attention calculation formula for the EfficientViT module is as shown in Equation (1)


(1) $$\begin{array}{c} Context_{i}=\sum_{j=1}^{N}\frac{Sim\left(Q_{i},K_{j}\right)}{\sum_{j=1}^{N}Sim\left(Q_{i},K_{j}\right)}V_{j}=\sum_{j=1}^{N}\frac{\phi \left(Q_{i}\right)\phi {\left(K_{j}\right)}^{T}}{\sum_{j=1}^{N}\phi \left(Q_{i}\right)\phi {\left(K_{j}\right)}^{T}}V_{j} \end{array}$$

In the formula:

$\left(Q,K,V\right)=xW_{(Q,K,V);}$

$Q_{i}$

—Row i of matrix Q;

$K_{j}$ —The j-th column of matrix K;

$V_{j}$ —The j-th column of matrix V;

$W_{\left(Q,K,V\right)}$ —Mapping matrix for learning;

ϕ(.) —Kernel function.

The EfficientViT network introduced in this text can enhance the recognition of subtle features and improve robustness in complex environments, by integrating multi-scale information and strengthening feature fusion, thereby further enhancing the model’s performance efficiency.

b) SIoU loss function

**Figure 6:**
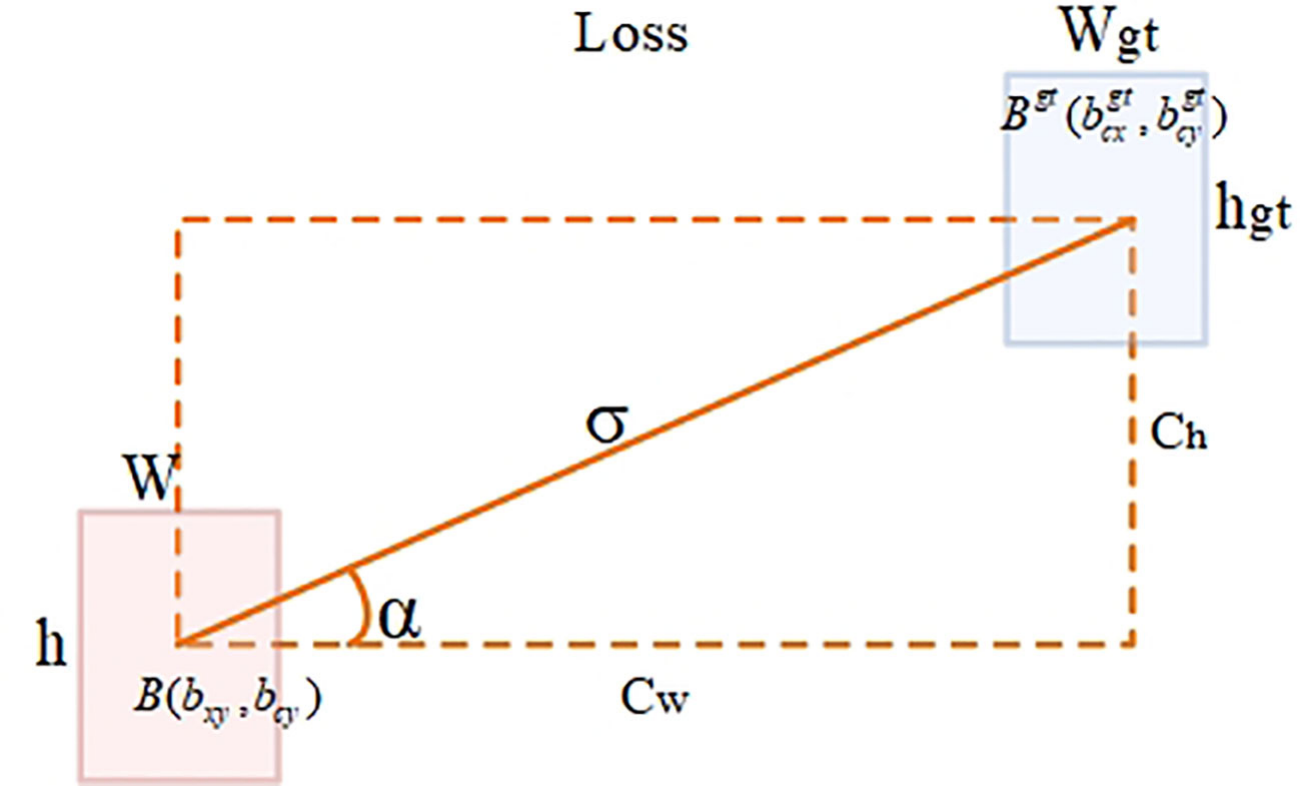
SIoU parameters schematic diagram. *B*( $b_{cx}$ , $b_{cy}$ )and $B^{gt}$ ( $b_{cx}^{gt}$ , $b_{cy}^{gt}$ ) represent the center coordinates of the predicted box and the ground truth box, respectively; C_w_ and C_h_ represent the differences in the horizontal and vertical coordinates between the *B* and $\;B^{gt}$ points, respectively; α is the horizontal angle between the center points of the two boxes; w, h, ^𝑤𝑔𝑡^ and ℎ^𝑔𝑡^ represent the width and height of the predicted box and the ground truth box, respectively; σ represents the distance between the center points of the ground truth box and the predicted box.

YOLOv8 uses the CIoU (Zheng et al., 2022) loss function to optimize localization loss. Although it considers the issues of aspect ratio and scale loss based on GIoU (Rezatofighi et al., 2019) and DIoU (Zheng et al., 2019), it relies on the aggregation of bounding box regression indicators. Due to the neglect of orientation mismatch issues, during training, the predicted boxes may affect the convergence speed and detection performance of the model due to “unordered wandering.” The SIoU (Gevorgyan, 2022) loss function (as shown in **Figure 6**) introduces the concept of vector angle, considers the angle issue between the true box and the predicted box, redefines the penalty metric, and improves the accuracy of the detection task. The SIoU loss function consists of four penalty terms: angle loss, distance loss, shape loss, and IOU loss.

The calculation formula for angle loss Λ is as shown in Equations (2), (3):


(2) $$\begin{array}{c} \Lambda =1-2{\text{sin}}^{2}\left(\arcsin\left(x\right)-\;\frac{\pi }{4}\right)\; \end{array}$$

(3) $$\begin{array}{c} x=\{\begin{array}{c} \sin\left(\alpha \right),\alpha \leq \frac{\pi }{4}\\ \sin\left(\beta \right),\alpha +\beta =\frac{\pi }{2}且\alpha >\frac{\pi }{4} \end{array}\; \end{array}$$

The formula for calculating the distance loss Δ is as shown in Equations (4), (5):


(4) $$\begin{array}{c} \rho_{x}={\left(\frac{b_{c_{x}}^{gt}-b_{c_{x}}}{c_{w}}\right)}^{2},\rho_{y}={\left(\frac{b_{c_{y}}^{gt}-b_{c_{y}}}{c_{h}}\right)}^{2} \end{array}$$

(5) $$\begin{array}{c} \Delta =\sum_{t=\text{x,y}}\left(1-e^{-\gamma \rho_{t}}\right)=2-e^{-\gamma \rho_{x}}-e^{-\gamma \rho_{y}} \end{array}$$

The shape loss Ω is defined as as shown in Equations (6), (7):


(6) $$\begin{array}{c} \Omega =\sum_{t=w,h}{\left(1-e^{-wt}\right)}^{\theta } \end{array}$$

(7) $$\begin{array}{c} \omega_{w}=\frac{\left|w-w^{gt}\right|}{\max\left(w,w^{gt}\right)},\omega_{h}=\frac{\left|h-h^{gt}\right|}{\max\left(h,h^{gt}\right)} \end{array}$$

In the formula, θ represents the weight of shape loss. θ∈[2,6] SIoU Loss Function is defined as shown in Equation (8):


(8) $$\begin{array}{c} L_{SIoU}=1-IOU+\frac{\Delta +\Omega }{2} \end{array}$$

c) C2f-Faster Module

The C2f module used in YOLOv8 enhances the image feature extraction capabilities, but the stacking of Bottleneck modules inevitably leads to redundancy in information channels and an increase in inference workloads. To address these issues, the Faster Block module was integrated into C2f, reducing both model computation and floating-point calculations (Chen et al., 2023). Partial Convolution (PConv) extracts features from only some channels of the input feature map, reducing redundant operations and memory access, thereby enhancing the capture of key spatial features. Assuming that the number of channels before and after outputting a feature map remains unchanged and that k is the kernel size, then PConv’s FLOPs per second (floating-point operations) and MAC (Memory Access Cost) calculation formula are as shown in Equations (9), (10):


(9) $$\begin{array}{c} FLOPs_{\left(PConv\right)}=h\times w\times k^{2}\times c_{p}^{2} \end{array}$$

(10) $$\begin{array}{c} MAC=h\times w\times 2c_{p}+k^{2}\times c_{p}^{2}\approx h\times w\times 2c_{p} \end{array}$$

**Figure 7:**
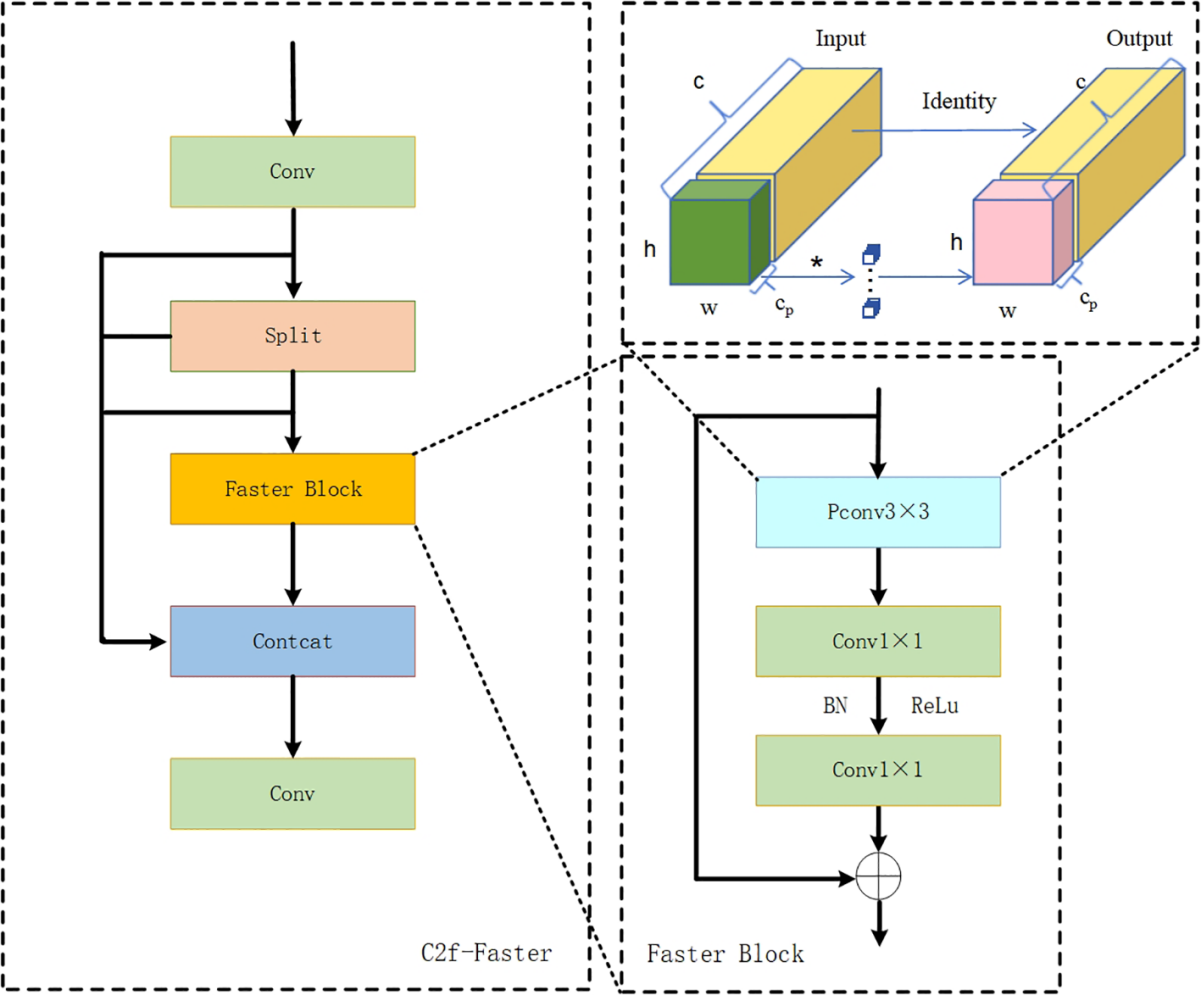
C2f-Faster module architecture diagram. h and w represent the height and width of the input feature map; c denotes the number of input channels; c_p_ denotes the number of channels participating in convolution; PConv stands for Partial Convolution; Split represents the channel splitting module.

This module performs convolution operations on a portion of the input channels, Cp, representing the entire feature map while keeping the remaining channels unchanged. Afterwards, it concatenates and overlays these processed channels with the remaining ones for output. Under a typical partial convolution rate (r=1/4), the computational cost of the improved C2f-Faster is approximately 1/16 that of C2f’s, featuring low memory occupancy during convolution and around 1/4 the memory access volume compared to regular convolutions. This design aims to reduce redundant computations, maximize channel information preservation, and enhance feature extraction. See **Figure 7** for the structural layout of C2f-Faster module.

d) Auxiliary detection head

In the YOLO series networks, the reduction of feature map size and resolution due to downsampling operations leads to the challenge of losing fine-grained information in learning complex image features. Therefore, this study introduces the strategy of Auxiliary Head from YOLOv7 (Wang et al., 2023). By embedding auxiliary heads in the middle layers of the network, additional gradient signals are provided to enhance gradient backpropagation. During the training process, the auxiliary detection head can extract more shallow network information, obtain fine-grained feature maps, and accelerate the regression of the loss function detection boxes. The introduction of auxiliary learning mode enhances the model’s understanding of multi-scale targets and complex scenes. Meanwhile, the auxiliary branch and the main classification branch merge to calculate the loss function, utilizing a richer gradient information flow to aid network training, thereby improving detection accuracy and reducing overfitting risks. Assuming α is the participation rate of the auxiliary detection head, the loss calculation for the auxiliary detection head is as shown in Equation (11):


(11) $$\begin{array}{c} LOSS_{\text{G}}=\alpha LOSS_{\text{A}}+(1-\alpha )LOSS_{\text{M}} \end{array}$$

In the formula: $LOSS_{\text{G}}$ — Total model loss;

$LOSS_{\text{A}}$ — Backbone network loss;

$LOSS_{\text{M}}$ — Loss of auxiliary detection heads.

### 2.4 Evaluation metrics

To measure the detection effects and performance between models, precision (Precision, P), recall rate (Recall, R), mean average precision (mean Average Precision, mAP), frames per second (Frames Per Second, FPS), model weight (MB), and floating-point operations (FLOPs) are selected as evaluation metrics to assess the final effect of the model (Jiang et al., 2018; S et al., 2023).

## 3 Results and analysis

### 3.1 Ablation experiments to improve the model

This study sets uniform training parameters and conducts 11 groups of ablation experiments aimed at accurately assessing the impact of various improvement strategies on multi-stage tomato detection. Given the needs for actual scenario detection, the YOLOv8n model is chosen as the baseline network. The model is evaluated through comparative metrics, with experimental results shown in **Table 1**.

**Table 1:** Results of ablation studies for the improved model.

No.	EfficientViT	C2f-Faster	SIoU	Aux-Head	PrecisionP/%	RecallR/%	Mean Average Precision mAP/%	Weight/MB	Floating Point Operations FLOPs/G	Frames Per Second FPS(frame/s)
1	×	×	×	×	80.5	77	86.7	5.99	8.1	114.94
2	√	×	×	×	89.7	88.5	93.6	4.63	6.3	107.53
3	×	√	×	×	88.1	83.5	88.8	6.58	8.9	171.59
4	×	×	√	×	85	88.1	91.6	5.97	8.1	107.53
5	×	×	×	√	89.2	84.9	89.4	7.45	8.1	103.09
6	×	√	√	×	83.6	80.2	86.8	4.67	6.7	97.09
7	√	×	×	√	91.9	88.5	93.5	9.24	9.4	99.01
8	√	√	×	×	93.3	87.7	94.9	6.37	8.7	101.01
9	√	×	√	×	87.9	82.3	88.7	5.34	9.4	106.38
10	√	√	√	×	88.1	84.1	91.1	7.8	8.7	167.10
11	√	√	√	√	91.4	88.7	93.9	8.06	9.4	163.33

“×” This policy is not used; “√” to use this policy.

According to the data in **Table 1**, Experiment 1 uses the original YOLOv8n model, achieving an accuracy of 80.5%, recall rate of 77%, and mAP of 86.7%, with a model weight of 5.99MB and 8.1GFLOPs of floating-point operations. Experiment 2, which replaced the backbone network with EfficientViT, shows increases in accuracy, recall rate, and mAP by 9.2%, 11.5%, and 6.9% percentage points respectively, compared to Experiment 1. This also results in a reduction in model weight and floating-point operations, indicating that the EfficientViT network significantly improves model performance by enhancing feature extraction capability and reducing the size and computational complexity of the model. Experiment 3 introduced C2f-Faster to optimize the feature transfer path and accelerate feature fusion, enhancing the model’s response speed, with accuracy and recall rates improving by 11.42% and 14.93% respectively; the frame rate increased by 49.29%. In Experiment 4, after replacing the SIoU loss function, the model’s accuracy, recall rate, and mAP all improved, suggesting that SIoU helps the model converge and enhances its recognition accuracy and stability. Experiment 5, which added an auxiliary detection head, led to a 2.7 percentage point increase in mAP, slightly improving detection accuracy. However, due to the addition of the detection head, the model weight increased by 1.46MB and the frame rate dropped by 10.31%. Compared to the baseline network, the improved model achieves optimal detection performance, with increases in accuracy, recall rate, and mAP of 10.9%, 11.7%, and 7.2% percentage points respectively. Although the introduction of more modules led to an increase in model weight and computational requirements, the detection performance significantly improved. Comprehensive ablation study results prove that the optimization strategies proposed for the YOLOv8n network in this study are meaningful.

### 3.2 Model performance comparison before and after improvements

**Figure 8:**
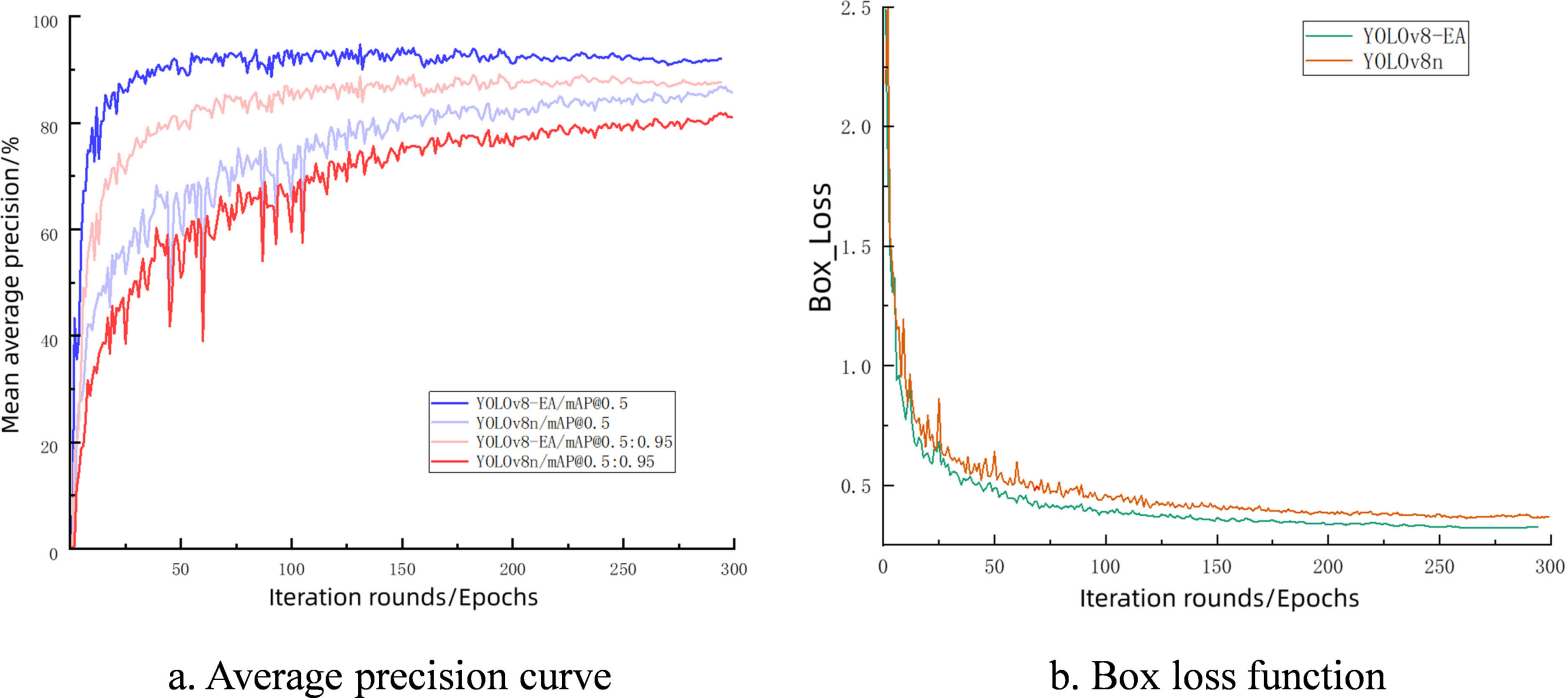
Training curves before and after model improvement. **(A)** Average precision curve **(B)** Box loss function.

**Figure 8** shows the comparison between the mean average precision (mAP) curves at different IOU thresholds and the box loss function for YOLOv8-EA and YOLOv8n. In **Figure 8A**, when the IOU threshold is 0.5, YOLOv8-EA shows a significant improvement in mean average precision compared to YOLOv8n. As the IOU threshold increases, the gap in accuracy performance between the two narrows, but YOLOv8-EA performs better across all IOU thresholds. This indicates that the YOLOv8-EA model has stronger predictive capability for bounding boxes. In **Figure 8B**, continuous declines in box loss reflect improvements in bounding box localization accuracies during training of both models, with YOLOv8-EA’s loss curve declining more rapidly which demonstrates its efficiency in learning bounding box localization; it consistently remains below that of YOLOv8n, showcasing stable training processes and superior convergence performance. Both in mAP curve or loss curve comparisons, fluctuations are less pronounced for YOLOv8-EA than forYOlov2 indicating enhanced learning capabilities improved stability of the revised model robustness.

The recognition performance of the improved model for multi-stage tomatoes is shown in **Table 2**. Compared to YOLOv8n, the improved YOLOv8-EA has increased the recognition accuracy of three stages of tomatoes by 10.1%, 17%, and 5.7% respectively, while increasing computational load by only 16%. This has resulted in an increase in detection precision mAP@0.5, mAP@0.5:0.95, and frame rate by 7.2, 7.5, and 81.25 percentage points respectively, providing powerful technical support for real-time tomato detection in complex environments.

**Table 2:** Test results before and after improvements of YOLOv8n model.

Stages	YOLOv8n				YOLOv8-EA			
	PrecisionP/%	Recall R/%	mAP@0.5	mAP@0.5:0.95	PrecisionP/%	Recall R/%	mAP@0.5	mAP@0.5:0.95
Unripe	85	85.8	0.923	0.871	95.1	93.4	0.971	0.915
Semi-ripe	67.2	67.6	0.784	0.72	84.2	88.8	0.91	0.86
Ripe	89.3	77.5	0.895	0.859	95	83.7	0.937	0.902

### 3.3 Comparison test of different detection models

To verify the effectiveness of the method discussed in this paper, it was compared with mainstream object detection algorithms on the same dataset, with results shown in **Table 3**. The results demonstrate that the improved YOLOv8-EA model surpasses other models in precision, recall rate, and average accuracy, proving that our enhanced model offers superior detection performance.

**Table 3:** Experimental results of different algorithms.

Models	Precisio P/%	Recall R/%	Mean Average Precision mAP/%	Weight/MB	Floating Point Operations FLOPs/G	Frames Per Second FPS
YOLOv5s	83.9	82.3	87.9	14.5	15.8	13.49
YOLOv7	80.7	76.2	84.2	74.8	103.2	53.19
YOLOv7-tiny	81.2	76.8	84.9	46.4	38.6	142.86
YOLOv8n	80.5	77	86.7	5.99	8.1	114.94
YOLOv8-EA	91.4	88.7	93.9	8.06	9.4	163.33

Additionally, the improved model features a frame rate detection that significantly surpasses other models. Even though this model has slightly larger weights and FLOPs compared to YOLOv8n, it still fits practical scenarios well. After comparing evaluation parameters, it is known that the improved model balances speed and efficiency effectively, exhibiting overall performance superior to other models especially in multi-stage fruit target detection.

**Figure 9:**
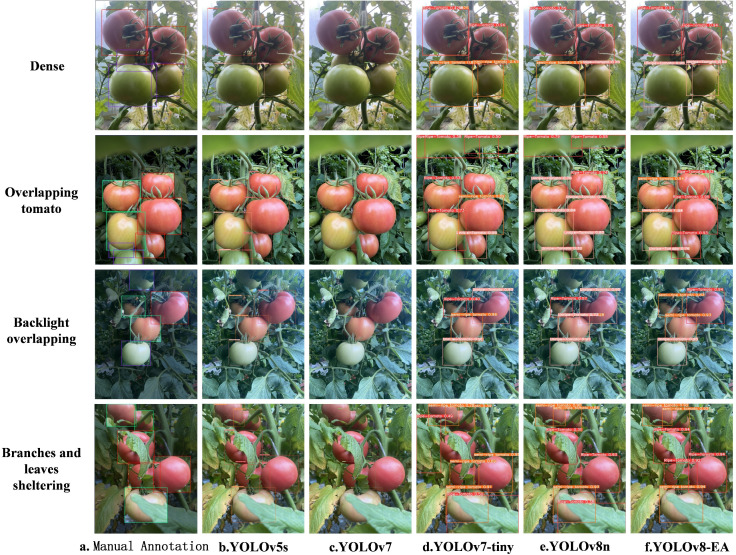
Comparison chart of detection effects. (i) Dense, (ii) Overlapping tomato, (iii) Back overlapping, (iv) Branches and leaves sheltering, **(A)** Manual Annotation. **(B)** YOLOv5s. **(C)** YOLOv7. **(D)** YOLOv-tiny. **(E)** YOLOv8n. **(F)** YOLOv8-EA.

**Figure 9** depicts the recognition effects of various mainstream target detection models on tomatoes at different growth stages. As observed from **Figure 8**, under complex conditions such as overlapping tomato fruits and occlusion by branches and leaves, other models exhibit instances of missed and false detections. However, the improved YOLOv8-EA model significantly ameliorates these issues. It shows enhanced performance in recognizing small target tomatoes in complex environments, with an increase also noted in confidence levels.

### 3.4 Comparative tests of different detection models on publicly available datasets

In order to conduct a comprehensive assessment of the enhanced YOLOv8-EA model, this study was subjected to evaluation using the publicly accessible dataset provided by Kaggle (http://www.kaggle.com). The dataset comprises a diverse range of real-world work scenarios, encompassing a total of 17,345 images that illustrate the various stages of tomato maturation. This makes it an optimal testing environment for validating the efficacy of each detection model.

Five mainstream detection models, including YOLOv8-EA, were selected for this test, and all models were completed under the same experimental platform to ensure the results were comparable and the process was fair and consistent. The principal performance metrics are illustrated in **Table 4**. The enhanced YOLOv8-EA model demonstrates robust performance on this public dataset, exhibiting a precision rate of 91%, a recall rate of 89.2%, and an average precision of 95.1%. These metrics demonstrate superior performance compared to other models, confirming the efficacy of optimising the model structure, particularly in the context of complex backgrounds and high-variability fruit images. Despite the increased weights and computational requirements of the YOLOv8-EA model, its detection speed can reach 1.8 ms, indicating that the model effectively optimises the utilisation of computational resources while maintaining high processing efficiency. Its exceptional performance renders it suitable for real-time processing scenarios where high efficiency and accuracy are paramount.

**Table 4:** Key performance indicators of different models on public datasets.

Models	Precision P/%	Recall R/%	Mean Average Precision mAP/%	Weight/MB	Floating Point Operations FLOPs/G	Detect_ time ms
YOLOv5s	92.2	90.4	92.8	17.7	15.8	18.6
YOLOv7	88.7	83.7	88.9	91.2	101.8	5.15
YOLOv7-tiny	89.2	84.4	89.6	56.57	38.6	2
YOLOv8n	87.5	85.6	91.5	7.3	8.1	2.2
YOLOv8-EA	91	89.2	95.1	11.2	10.7	1.8

## 4 Discussion

We reviewed previous related research work, based on which we proposed the YOLOv8-EA model for detecting multi-stage tomato fruits, taking into account the differences between actual tomato picking conditions and individual fruit ripening stages. The previous section 3 demonstrates its remarkable performance and accuracy.

EfficientViT employs sandwich-layout blocks, using a single memory-efficient MHSA between effective feed-forward networks (FFN), enhancing storage efficiency while increasing the number of feature channels. It introduces a new Cascaded Group Attention module (CGA), which maximizes computational cost savings while ensuring high-quality key feature extraction; SIoU evaluates the overlap between predicted and ground truth boxes more reasonably, enabling the model to reach its optimal state more quickly during training; PConv exploits the redundancy in feature maps by systematically applying regular convolution (Conv) on a subset of input channels without affecting others. Additionally, a pointwise convolution (PWConv) is added on top of PConv to fully and effectively utilize information from all channels. This approach reduces the number of parameters and computational complexity while maintaining a certain receptive field and nonlinear representation capability; Aux-Head provides additional supervision signals at the early stages of training, enhancing feature extraction capabilities and thereby improving overall detection accuracy. This richer information feedback stream accelerates model convergence and alleviates memory pressure. Aux-Head is used to capture shallow network information, employing Detect to guide Aux-Detect in matching positive detection samples, which addresses performance degradation and poor positive sample quality issues as model depth decreases. Therefore, the YOLOv8-EA detection model has both lightweight and high detection performance.

Despite the improvements we have made, which have significantly enhanced the model’s performance and accuracy, there are still some limitations that need to be addressed. These issues warrant deeper exploration in future work. First, the introduction of the EfficientViT module and the C2f-Faster module has reduced the model’s parameters and computational complexity, accelerating its running speed. However, further optimization of the model is still needed in future work. Second, although the new loss function speeds up the model’s convergence, the accuracy of bounding box localization may still be insufficient in cases of complex edges or significant overlap of target objects. For severely occluded fruits and scenes with significant lighting variations, the recognition efficiency and accuracy decrease, necessitating further research to improve the loss function or introduce newer feature extraction and fusion techniques. Furthermore, while the auxiliary detection head (Aux-Head) module enhances the network’s learning capability, it also increases the model’s structural complexity. This means that more computational resources and storage space are required during model training and deployment, which could pose challenges for deployment on resource-constrained edge devices. Lastly, the model proposed in this study performs excellently on the tomato dataset, but its generalization ability to other crop datasets remains to be verified.

## 5 Conclusion

This paper is based on the YOLOv8-EA multi-stage detection model for tomatoes, achieving rapid and accurate detection of tomato fruits in complex environments. It also validates the improved model’s detection performance on a homemade dataset, with the main conclusions as follows:

1) The architecture adopts the EfficientViT network as the backbone, introduces the SIoU loss function and C2f-Faster module, along with additional optimized strategies such as auxiliary detection heads. On the self-constructed dataset, compared to the baseline network YOLOv8n, with only a 2.07MB increase in model weight and a 1.3G rise in FLOPs, accuracy improvements for detecting unripe, semi-ripe, and ripe tomatoes have respectively increased by 4.8%, 12.6%, and 4.2% points; meanwhile, the frame rate of detection has improved by 42.1%, achieving enhancements in both detection efficiency and precision.

2) Whether on the self-built dataset or the open dataset, compared with the current mainstream target detection models, the YOLOv8-EA model proposed in this study outperforms other models in a number of indexes, with obvious advantages in the comprehensive performance, and has a better detection effect on multi-stage tomatoes, providing technical support for the subsequent intelligent picking.

3) Through a visual comparison of detection results, YOLOv8-EA shows fewer missed and false detections of tomatoes in complex environments, providing optimal detection ability. This indicates the feasibility of the proposed object detection algorithm. Subsequent efforts will further optimize the model’s parameter volume to adapt to practical environments with limited computing resources.

## Data Availability

The original contributions presented in the study are included in the article/supplementary material. Further inquiries can be directed to the corresponding author.
